# Emotional Distress and Cardiovascular Health in Young Adults with Type 1 Diabetes

**DOI:** 10.3390/jcdd11120391

**Published:** 2024-12-05

**Authors:** Bethany L. Armentrout, Bootan H. Ahmed, Sineenat Waraphok, Johnathan Huynh, Stephanie Griggs

**Affiliations:** Frances Payne Bolton School of Nursing, Case Western Reserve University, Cleveland, OH 44106, USA; bla31@case.edu (B.L.A.); bha25@case.edu (B.H.A.); sxw1243@case.edu (S.W.); johuynhofficial@gmail.com (J.H.)

**Keywords:** distress, diabetes distress, young adult, type 1 diabetes, cardiovascular health

## Abstract

Type 1 diabetes (T1D) is a complex chronic condition that places young adults aged 18–31 years at high risk for general and diabetes-related distress and poor cardiovascular health. Both general and diabetes distress are linked to higher A1C, a known risk factor for cardiovascular disease (CVD). The purpose of this cross-sectional quantitative descriptive study was to examine the associations between distress symptoms (general and diabetes) and cardiovascular health while considering covariates in young adults ages 18–31 years with T1D. One-hundred and sixty-five young adults with T1D, recruited from specialty clinics through two major health systems and online platforms, completed a demographic and clinical survey along with the 8-item PROMIS Emotional Distress Scale and 17-item Diabetes Distress Scale. Higher diabetes distress and higher general emotional distress were associated with lower cardiovascular health scores. Associations remained statistically significant after adjusting for age, T1D duration, sex at birth, race, and continuous subcutaneous insulin infusion. In young adults with type 1 diabetes, addressing both diabetes and general emotional distress may be important to improve cardiovascular health. However, longitudinal and experimental studies are needed to clarify underlying mechanisms and evaluate the effectiveness of interventions like cognitive behavioral therapy.

## 1. Introduction

The 2 million individuals living with type 1 diabetes (T1D) have a threefold higher cardiovascular disease (CVD)-related mortality risk, occurring 10–15 years earlier when compared to the general United States population [1,2]. Despite the overall improvement in CVD-related mortality in the United States, the proportion of young adults in the general population with CVD-related mortality has increased [3,4]. Further, there is a CVD incidence of 1.2% per year among all adults with T1D, including young adults [5,6,7]. In three large cohorts of adults with T1D, including the Allegheny County (*n* = 1075), Norwegian (*n* = 1096), and Diabetes U.K. Study (*n* = 23,752), before age 30 years, acute metabolic complications were the leading cause of mortality; however, after the age of 30 years, CVD-related mortality predominated [8,9,10]. Cardiovascular health (CVH), comprised of four health behaviors (diet, nicotine exposure, physical activity, and sleep) and four health factors (cholesterol, glucose, body mass index, and blood pressure), is associated with a markedly lower risk for CVD events [11]. Only one-third of the general young adult population achieves ideal CVH [12]. Although the prevalence of ideal CVH status among young adults with T1D is unknown, a majority (75–83%) do not meet the recommended glycated hemoglobin (A1C) targets (A1C < 7%) [13,14,15].

Psychological distress is associated with earlier CVD onset, more rapid CVD progression, and increased CVD-related mortality [16,17,18,19]. This connection is particularly pronounced among young adults with T1D who are at high risk for general and diabetes-related psychological distress (diabetes distress) as they navigate a complex landscape of multiple life transitions related to housing, finance, insurance, education, and career development [13,14]. Further, psychological distress is associated with many life transition factors and further complications of diabetes such as retinopathy neuropathy and nephropathy [13,14]. Interventions targeting psychological distress, such as cognitive behavioral therapy (CBT), offer a promising pathway to mitigate these risks. CBT has not only been effective in alleviating symptoms of anxiety and depression, which are prevalent among individuals with T1D, but it has also shown marked improvements in individual CVH indicators, notably in hemoglobin A1C levels and low-density lipoprotein (LDL) cholesterol levels [20,21,22]. The underlying mechanism by which CBT enhances cardiovascular health, primarily through the reduction of psychological distress, cannot be overstated. Psychological distress has been shown to exacerbate physiologic responses such as chronic inflammation, endothelial dysfunction, and dysregulation of autonomic functions—all of which contribute to CVD risk [16]. By effectively targeting and alleviating this distress, CBT not only fosters better mental health outcomes but also potentially mitigates these harmful physiologic responses.

While existing studies have established the negative impact of psychological factors on health outcomes in the general population, they often overlook the unique experiences and challenges faced by young adults with T1D. For instance, in a study with 83 middle-aged adults (mean age 45 years, mean T1D duration 20 years), higher diabetes distress was significantly associated with one individual CVH factor, higher A1C. In contrast, the other individual CVH factors (blood pressure, total cholesterol, and body mass index) or behaviors (physical activity and smoking) measured in the study were not significant [23]. The existing literature is focused on middle- or older-aged adults with T1D and the general and diabetes-related stress–individual CVH factor association, predominantly A1C and/or behaviors. Given that individuals with T1D face a shortened life expectancy, it is crucial to assess CVH earlier in life. Although both general and diabetes-related stress have been explored in those with T1D, there is limited evidence regarding their contribution to CVH, especially during young adulthood—an age marked by significant life transitions and events.

**Figure 1:**
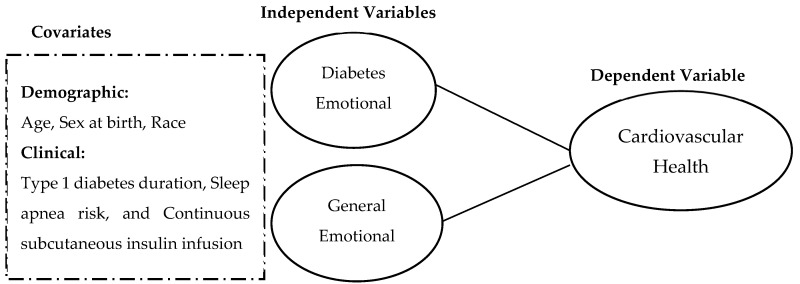
Conceptual model of distress and cardiovascular health in young adults with type 1 diabetes.

Therefore, the purpose of this quantitative descriptive cross-sectional study is to examine the associations between distress symptoms (general and diabetes) and a CVH composite while considering covariates in young adults aged 18–31 years with T1D living in the United States. Emotional and diabetes-related symptoms are potentially modifiable targets that if addressed may improve CVH in this population achieving cardiovascular targets at the lowest rates (e.g., young adults with T1D) (Figure 1).

## 2. Materials and Methods

### 2.1. Study Design

The procedures were performed in accordance with the Declaration of Helsinki and were approved by the Institutional Review Boards (STUDY20200650; STUDY20201829; STUDY20211165). We conducted a cross-sectional analysis from three cohorts of young adults with T1D recruited via convenience sampling from the United States with data collected 2018–2019 (diabetes specialty clinics in Northeast), 2021–2022 (internet-based national), and 2022–2023 (diabetes specialty clinics in Midwest) from each cohort, respectively. Methods on these cohorts have been reported previously and details pertinent to this study are described in the sections below.

### 2.2. Participants

We included young adults who (1) were between 18 and 31 years of age; (2) had T1D for at least 6 months; (3) had no other complex comorbid medical (e.g., active cancer) or severe psychiatric condition (e.g., schizophrenia or bipolar disorder); and (4) were not enrolled in an intervention study. We screened out participants for the following reasons: (1) a known sleep disorder diagnosis including obstructive sleep apnea, periodic limb movement disorder, restless legs syndrome; (2) current pregnancy; or (3) night shift or recent trans-meridian travel.

### 2.3. Covariates

Covariates including age (years), T1D duration (years), race (white/non-white), sex at birth (male or female), continuous subcutaneous insulin infusion (insulin pump), and sleep apnea risk were chosen a priori based on the extant literature [18,19,20,21,22,23,24,25].

### 2.4. Emotional Distress Symptoms

General emotional distress was measured with the 8-item PROMIS v1.0 (emotional distress-depression) with a previous Cronbach’s alpha of 0.95 [24] in a general population and 0.88 in a similar population with T1D [25]. Items were ranked using a 5-point Likert scale (ranging from never to always) [24]. Raw scores were standardized into a T-score with T-scores ranging from 38.2 to 81.3 [24]. The Cronbach’s alpha in the current study was 0.950.

Diabetes emotional distress was measured with the 17-item Diabetes Distress Scale (DDS) with a previous Cronbach’s alpha ranging from 0.88 to 0.93 in primary care diabetes populations (type 1 and type 2 diabetes) from four clinical sites in the United States) [26]. The DDS measures diabetes-related emotional distress, and each item was answered using a 6-point Likert scale (1 = not a problem to 6 = a very serious problem) reflecting the degree to which the item is perceived as a problem [26]. Total scale scores range from 17–102, with higher scores indicating higher diabetes-related emotional distress [26]. The mean-item scores range from 1 to 6. The total DDS score was used in the regression analysis to capture diabetes-related distress as a whole [26]. The Cronbach’s alpha in the current study was 0.928.

### 2.5. Cardiovascular Health

A CVH composite was derived from four behaviors (diet, physical activity, nicotine exposure, and sleep) and two factors (body mass index and glycemia) [27]. Each individual component was graded on a 0 to 100 scale and then divided by the number of components to quantify an overall CVH score. Each item was scored according to the most recently published AHA recommendations. Sleep health (7–<9 h = 100, 9–<10 h = 90, 6–<7 h = 70, 5–<6 h = 40, 4–<5 h = 20, <4 h = 0), nicotine exposure (100 = nonsmoker, 0 = smoker), diet (recoded scores 30 = 100, 25–29 = 80, 20–24 = 50, 13–19 = 25, 6–12 = 0), and physical activity (recoded 3 = 100, 2 = 80, 1 = 60, 0 = 0). The four CVH behaviors were derived from the baseline survey across the studies, including multi-item physical activity, diet subscales, one sleep duration item from the Pittsburgh Sleep Quality Index, and one nicotine item (smoking yes/no) [28,29]. Most recent glycated hemoglobin (A1C) values were used for glycemia and weight in kilograms and height in centimeters were used to calculate the body mass index (kg/m^2^). A1C (4–6.9 = 40, 7–7.9 = 30, 8–8.9 = 20, 9–9.9 = 10, 10–highest = 0) and BMI (17–24.9 = 100, 25–29.9 = 70, 30–34.9 = 30, 35–39.9 = 15, 40–highest = 0).

### 2.6. Statistical Analysis

We managed the data on Research Electronic Data Capture (REDCap) (hosted at Yale University, Case Western Reserve University, and University Hospitals, Cleveland) [30,31] and analyzed the data via the Statistical Package for the Social Sciences version 29.0.2.0 for Mac. Descriptive statistics were used to summarize each variable, including multi-item scale scores. Prior to analysis, data were screened for missing or implausible values and distributions of continuous variables through visual inspection and an assessment of descriptive statistics including skewness and kurtosis values. A series of t-tests, one-way analyses of variance, and correlational analyses were used to explore possible differences in sex, race, age, and T1D duration in the variables of interest. To evaluate the explanatory contributions of distress (general and diabetes) to the CVH composite, we performed a series of multivariable linear regression models. Separate models were examined with general and diabetes distress as the predictors. Linear regression models were adjusted for covariates, age, sex, race (white, non-white), sleep apnea risk (high/low), T1D duration, and continuous subcutaneous insulin infusion (yes/no). Statistical significance was set at *p* < 0.05. In assessing model fit, the predicted value mean indicated a well-fitting model with minimal variability. Multicollinearity was not detected as the tolerance and VIF values were 0.912 and 1.096 for general distress and 0.823 and 1.216 for diabetes distress, respectively, indicating independence among predictor variables.

## 3. Results

### 3.1. General Characteristics

One hundred and sixty-five young adults with T1D were included in the present study. Participants’ mean age was 21.5 (±2.5) years, mean BMI was 25.6 (±4.9) kg/m^2^, and a little over half (54.9%) were in college. A majority were female (59.5%), Non-Hispanic White (77%), and diagnosed with T1D during their childhood or adolescent years with a mean diabetes duration of 10.3 ± 5.6 years. The demographic and clinical characteristics of the overall sample are presented in Table 1 and Table 2.

### 3.2. General and Diabetes Emotional Distress

We present clinical characteristics in Table 2. The mean general emotional distress t score for the total sample was 52.5 (±9.6). A quarter (22.0%, *n* = 36) met the cut-off for moderate general emotional distress (standardized t score +10 above the standard 50). The mean diabetes distress total score for the sample was 36.3 (±15.7) and the mean-item score was 2.1 (±0.92). Forty-eight participants (31%) reported moderate diabetes distress (mean item score 2.0–2.9) and twenty-six (16.8%) met the threshold for high diabetes distress (mean item score ≥ 3.0).

There were sex differences in reported diabetes distress symptoms. Specifically, females reported higher diabetes distress (*M* ± *SD* = 41.91 ± 17.48 vs. 33.11 ± 13.92 *p* = 0.025). There were no significant sex differences in general emotional distress. Older individuals had a lower A1C (r = −0.287, *p* = 0.016). Individuals identifying as Non-White had a lower cardiovascular health score than individuals identifying as White ((*M* ± *SD* = 64.9 ± 14.31 vs. 71.86 ± 18.35 *p* = 0.044). Those with a high sleep apnea risk had a lower cardiovascular health score (*M* ± *SD* = 60.73 ± 14.04 vs. 71.98 ± 17.87 *p* = 0.009).

### 3.3. Associations Between Diabetes Emotional Distress and Cardiovascular Health

First, we examined bivariate associations between diabetes emotional distress and CVH. Higher diabetes emotional distress was associated with lower CVH scores (*r* = −0.165, *p* = 0.034). Next, the contribution of diabetes emotional distress to CVH was tested in a standard linear regression model (Table 3). The association between diabetes distress remained statistically significant (*β* = −0.298, *p* = 0.004) after controlling for covariates (age, sex at birth, race, sleep apnea risk, T1D duration, and continuous subcutaneous insulin infusion) accounting for 20.9% of the variance. Additionally, older age was significantly associated with a higher cardiovascular health composite score.

### 3.4. Associations Between General Emotional Distress and Cardiovascular Health

Higher general emotional distress was associated with lower cardiovascular health scores in the bivariate correlation model (*r* = −0.198, *p* = 0.011). Next, we examined the contribution of general emotional distress to CVH (Table 4). The association between general emotional distress and CVH remained statistically significant (*β* = −0.335, *p* < 0.001) after controlling for covariates accounting for 23.9% of the variance. Additionally, a lower sleep apnea risk was associated with higher CVH.

## 4. Discussion

Among young adults with T1D, both diabetes distress and general emotional distress symptoms were inversely associated with cardiovascular health even when controlling for age, T1D duration, sex at birth, race, sleep apnea risk, and continuous subcutaneous insulin infusion. Our findings highlight the need to consider both diabetes and general emotional distress symptom severity in CVH among young adults with T1D. Specifically, understanding the prevalence of distress and overall CVH within this population can provide insight into the broader implications for CVH in this population.

In the current study, about one-third (54.7%) met the criteria for moderate general emotional distress, one-quarter (22%) met the moderate diabetes distress criteria, and one-third (31%) met the high diabetes distress criteria. These findings were consistent with comparable studies [23,26,32,33]. To illustrate, in one study of 74 participants with T1D (mean age 45.2 ± 16.6, A1c 8.78.7 ± 1.6), 27% reported moderate diabetes distress [23]. Additionally, female participants in the current study reported higher diabetes distress, which is consistent with other diabetes distress studies in adolescents and adults living with T1D [28,29]. However, there were no significant sex differences in general emotional distress in the current study.

In the current study, we found that both higher diabetes distress and general distress were associated with lower CVH, a result consistent with findings from other research [23,34]. While our hypothesis was supported, the physiological mechanisms underlying this are complex. One key mechanism involves the role of glucocorticoids, steroid hormones that are released during both physiological and psychological stress. These hormones are crucial for maintaining glucose homeostasis [35] and are vital in regulating energy metabolism, inflammation, and cardiac function [36]. Specifically, glucocorticoids promote hepatic gluconeogenesis [37,38] while simultaneously reducing glucose uptake and utilization in skeletal muscle and white adipose tissue [39]. During stress—such as fasting or starvation—the metabolic adaptation process preserves plasma glucose levels, ensuring the brain has a reliable energy source [35,40]. This transient increase in glucose can be beneficial for maximizing brain function [35,40]; however, this process can be particularly damaging to the vasculature. Additionally, the lack of insulin–glucose regulation in people with T1D raises the risk of affecting multiple systems, thereby increasing the overall risk of CVD.

Some limitations should be considered when interpreting the results of the current study. First, the current study was cross-sectional and designed to assess the associations between self-reported emotional distress and CVH behaviors and factors; therefore, we cannot elucidate the directionality of these associations. Future studies with objective measures (e.g., actigraphy for physical activity and sleep) could help validate the findings. In addition, there may have been some unmeasured covariates or confounders (e.g., insulin dose, sleep apnea through polysomnography, or central vs. peripheral obesity indicators such as waist circumference) contributing to the variance in cardiovascular health factors and or behaviors. Second, the self-reported data are subject to social desirability bias and respondents may have overestimated sleep duration and physical activity or underestimated nicotine exposure and healthy diet patterns according to previous research [41,42]. Further, two CVH factors (blood pressure and cholesterol) were unmeasured in this population. Third, the current study does not address CVH status for recent cardiovascular events, nor does it account for clinical diabetes symptoms and complications (e.g., retinopathy, neuropathy, and nephropathy) nor recent hospitalizations. Lastly, the sample composition (77% Non-Hispanic White and 59.5% female, age range 18–31 years) limits the generalizability to the broader T1D population, including other age groups or clinical populations with sleep disorders. Due to the limited representativeness, we were not able to correlate socioeconomic factors shown to increase stress with CVH.

There are also several strengths to help offset these weaknesses. For instance, we included a more comprehensive measure of CVH as conceptualized by the American Heart Association. Previous studies focused on individual factors and behaviors, limit our understanding of CVH. The inclusion criteria, which specify a narrow age range of 18–31 years and exclude individuals with other complex medical or psychiatric conditions, reduce the variability attributable to these confounding factors, thereby enhancing this study’s internal validity. Additionally, investigating complex, multivariable relationships beyond correlation strengthens the understanding of relationships among the study variables, including various covariates.

Though we and other teams have hypothesized emotional distress as a predictor of CVH, the direction cannot be inferred in the current study nor the comparison studies due to the cross-sectional nature of the designs. In another study, A1C and microvascular complications were found to predict diabetes distress in T1D, as measured by a comparable distress scale, the Problem Areas in Diabetes (PAID) [43]. It is possible that A1C levels may predict diabetes distress or that the relationship is reciprocal. In that case, internal self-regulatory mechanisms may influence these changes and future focus should be placed on understanding the psychological, financial, and physiological drivers of CVH factors and behaviors and resultant CVD in this population.

The mechanisms of internalized problems (e.g., stress arousal, emotion processing, and cognitive factors) underlie several modifiable risks [44]. In this regard, distress symptoms are malleable. They may be amenable to cognitive behavioral interventions (CBT), as evidenced by several randomized control trials (RCT) and pilot studies, including CBT interventions showing improvements in psychological and clinical outcomes in T1D [20,21,22]. Specifically, CBT has shown some promise in improving two key CVH factors, including A1C and low-density lipoprotein levels (LDL) [20,21,22,45]. Further, in a systematic review and meta-analysis of 19 RCT studies, it was reported that longer-term CBT for 6 months significantly lowered A1C [46,47]. However, there was no significant effect on A1C for those in shorter-term treatment (less than 6 months) [46,47]. Therefore, implementing CBT for at least 6 months could improve the psychological symptoms as well as clinical outcomes in young adults with T1D [45].

In young adults with T1D, both diabetes-related and general emotional distress were associated with lower CVH, highlighting the need to address these psychological factors in clinical care. However, while these associations are informative, they do not elucidate the underlying mechanisms in these associations. To fully understand how emotional distress impacts overall CVH and specific factors such as A1C, blood pressure, and cholesterol levels, further experimental studies are essential. These investigations should also aim to uncover the physiological pathways involved and assess the effectiveness of cognitive behavioral therapy (CBT) in alleviating psychological distress and improving CVH. It remains uncertain whether CBT interventions yield short-term clinical benefits—such as reduced glucose variability or lower A1C levels—or have a more significant impact on long-term clinical outcomes and complications, both micro- and macrovascular. Enhancing our understanding of these complex associations could lead to targeted strategies that improve both emotional and cardiovascular health for young adults living with T1D.

## Figures and Tables

**Table 1:** Sample demographic characteristics.

Characteristic	Mean	SD
Age in years	21.5	2.5
T1D Duration in years	10.3	5.6
	**N**	(**%**)
Education (% college student)	90	54.9
Sex at birth (female)	104	63
**Gender identity**		
Woman or female	97	59.5
Man or male	44	27.0
Trans-male	1	0.6
Genderqueer	2	1.2
Non-binary	4	2.5
**Self-reported race and ethnicity**		
Non-Hispanic White	124	77
Non-Hispanic Black	11	6.8
Non-Hispanic Asian	8	5.0
Hispanic White	9	5.6
Hispanic Black	3	1.9
Bi/Multi race/ethnicity	6	3.7

**Table 2:** Sample Clinical Characteristics.

Characteristic	Mean	SD
Emotional Distress		
Diabetes Emotional Distress Scale	36.3	15.7
General Emotional Distress (PROMIS t score)	52.5	9.6
**Cardiovascular Health**		
Total Cardiovascular Health Score	65.5	16.3
	**N**	**%**
Ideal Cardiovascular Health	33	20.6
Intermediate Cardiovascular Health	105	65.6
Poor Cardiovascular Health	22	13.8

Diabetes Emotional Distress Scale ranges from 17–102 with little or no distress defined as mean item scores < 2.0; moderate distress defined as mean item scores 2.0–2.9; and high distress with mean item scores ≥ 3.0. PROMIS: Patient-Reported Outcomes Measurement Information System, Total Cardiovascular Score range from 0 to 100; Ideal Cardiovascular Health 80–100, Intermediate Cardiovascular Health 50–79, and Poor Cardiovascular Health 0–49.

**Table 3:** Linear regression model of diabetes emotional distress to cardiovascular health composite.

Independent Variables	B	SE	β	*p* Value	R^2^
Diabetes Emotional Distress	**−0.293**	**0.099**	**−0.301**	**0.004**	0.210
Age	**1.799**	**0.754**	**0.229**	**0.019**	
Sex at birth	2.287	3.452	0.068	0.509	
Race	5.242	4.226	0.121	0.218	
Sleep apnea risk	−9.005	4.901	−0.187	0.069	
T1D Duration	−0.288	0.298	−0.099	0.336	
Continuous subcutaneous insulin infusion	−2.412	3.567	−0.069	0.501	

Note. B is the unstandardized coefficient regression coefficient. SE standard error. β is the standardized regression coefficient. R^2^ = coefficient of determination shown for each model. Significant values are bolded. Covariates included age, sex at birth, race (white/non-white), sleep apnea risk, type 1 diabetes duration, and continuous subcutaneous insulin infusion (yes/no).

**Table 4:** Linear regression model of general emotional distress to cardiovascular health composite.

Independent Variables	B	SE	β	*p* Value	R^2^
General Emotional Distress	**−0.543**	**0.152**	**−0.338**	**<0.001**	0.239
Age	1.382	0.746	0.176	0.067	
Sex at birth	0.876	3.235	0.026	0.787	
Race	5.037	4.148	0.117	0.228	
Sleep apnea risk	**−9.505**	**4.731**	**−0.197**	**0.047**	
T1D Duration	−0.331	0.293	−0.114	0.262	
Continuous subcutaneous insulin infusion	−1.280	3.496	−0.037	0.715	

Note. B is the unstandardized coefficient regression coefficient. SE standard error. β is the standardized regression coefficient. R^2^ = coefficient of determination shown for each model. Significant values are bolded. Covariates included age, sex at birth, race (white/non-white), sleep apnea risk, type 1 diabetes duration, and continuous subcutaneous insulin infusion (yes/no).

## Data Availability

Data are available upon reasonable request.
